# Empyema Necessitans Complicating Pleural Effusion Associated with *Proteus* Species Infection: A Diagnostic Dilemma

**DOI:** 10.1155/2015/108174

**Published:** 2015-03-29

**Authors:** M. S. Yauba, H. Ahmed, I. A. Imoudu, M. O. Yusuf, H. U. Makarfi

**Affiliations:** ^1^Department of Paediatrics, University of Maiduguri College of Medical Sciences, Maiduguri 752106, Nigeria; ^2^Department of Paediatrics, Federal Medical Centre, Azare 751101, Nigeria

## Abstract

*Background*. Empyema necessitans, a rare complication of pleural effusion, could result in significant morbidity and mortality in children. It is characterized by the dissection of pus through the soft tissues and the skin of the chest wall. *Mycobacterium tuberculosis* and *Actinomyces israelii* are common causes but Gram negative bacilli could be a rare cause. However, there were challenges in differentiating between *Mycobacterium tuberculosis* and nontuberculous empyema in a resource poor setting like ours. We report a child with pleural effusion and empyema necessitans secondary to *Proteus* spp. infection. *Methods*. We describe a 12-year-old child with empyema necessitans complicating pleural effusion and highlight management challenges. *Results*. This case was treated with quinolones, antituberculous drugs, chest tube drainage, and nutritional rehabilitation. *Conclusion*. Empyema necessitatis is a rare condition that can be caused by Gram negative bacterial pathogens like *Proteus* species.

## 1. Introduction

Empyema necessitans is a rare long-term complication of poorly or uncontrolled empyema thoracis characterized by the dissection of pus through the soft tissues and skin of the chest wall [1]. The pus collection bursts and communicates with the exterior, forming a fistula between the pleural cavity and the skin [1]. Pleural effusion with empyema necessitans is usually caused by* Mycobacterium tuberculosis *and* Actinomyces israelii* [2]. The most common nontubercular etiological agent is* Staphylococcus* [3]. Other microbial causes include Pneumococci,* Escherichia coli*,* Pseudomonas*,* Klebsiella*, and anaerobes [3]. Pleural fluids are usually diagnostic and help in the choice of appropriate antibiotics. However, it is very difficult to differentiate tuberculous from a nontuberculous empyema, especially in malnourished children and resource poor countries, because of the difficulty in diagnosing tuberculosis in children and lack of modern facilities for diagnosis of tuberculosis. Further investigations and management depend on the stage of the disease. Treatment of this condition would include antibiotics, tube drainage, and decortication for obliterating the cavity and regenerating pulmonary function.

## 2. Case Presentation

This is a 12-year-old boy who presented with low grade fever and cough for 3-month duration and chest pain for 7-week duration. Cough was insidious in onset and productive of purulent and nonbloody sputum. No history of contact with tuberculosis or chronically coughing adult. Seven weeks prior to presentation, he developed right sided dull aching chest pain that was nonradiating. There was associated difficulty in breathing but no discoloration of the mucous membrane. Fifteen days before presentation, he developed a swelling on the right side of the chest wall which became fluctuant and later ruptured and started discharging foul smelling pus. Appetite had been good but there was associated weight loss. There were no other systemic symptoms and he was not a known sickle cell anaemia subject. Developmental and nutritional history was uneventful. He was only given oral and topical traditional concoction at home with no relief of symptoms and the past medical history was not significant. He has not had any vaccination due to sociocultural factors.

**Figure 1:**
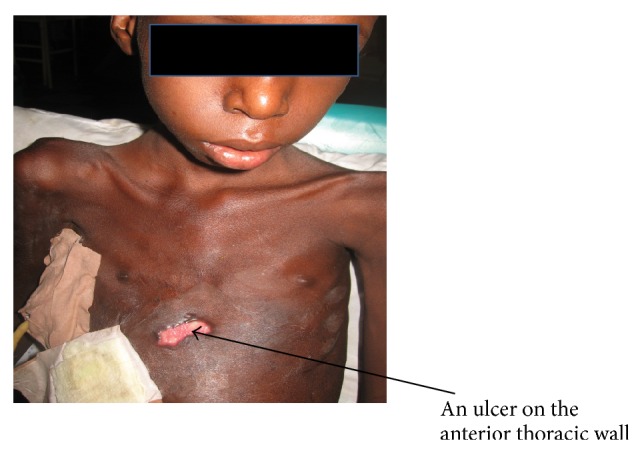
A picture of the child showing empyema necessitans.

**Figure 2:**
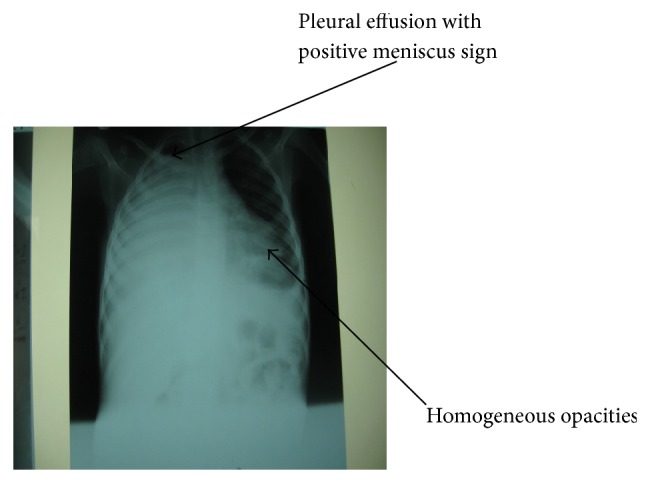
Chest X-ray showing pleural effusion with consolidation.

**Figure 3:**
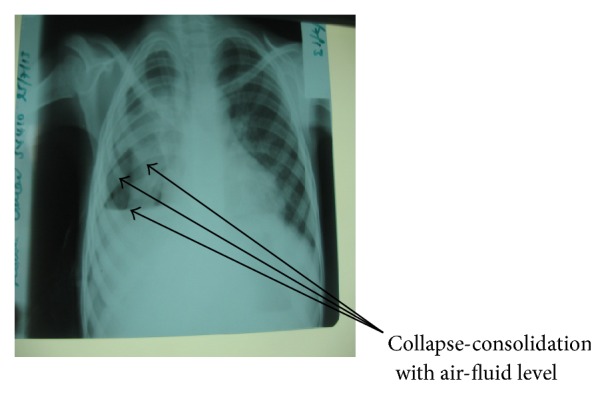
A chest X-ray after two-week course of antibiotics.

On examination he was found to be chronically ill-looking, wasted, and stunted, with a *Z*-score of <−3 standard deviation according to the World Health Organization classification of malnutrition. He was febrile (37.8°C), severely pale, and in obvious respiratory distress and had significant axillary lymphadenopathy. Respiratory system examination revealed flattening of the right chest wall with a purulent discharging tender ulcer with necrotic base on the right side of the chest wall. He was tachypneic with respiratory rate of 44 cycles/min. He was also dyspnoeic with reduced chest expansion on the right hemithorax. There was a stony dull percussion note on the right hemithorax but dull percussion notes on the left hemithorax. There was markedly reduced breath sounds intensity on the right hemithorax with widespread crepitation. He had tachycardia of 140 beats/min with displaced apex beat and grade two haemic murmurs. There was soft tender hepatomegaly of 4 cm below the right costal margin. Another systemic examination was normal. An initial diagnosis of pleural effusion with empyema necessitans secondary to pulmonary tuberculosis in anaemic heart failure was made (Figure 1). Chest X-ray showed right sided pleural effusion with homogeneous opacity and left sided opacities (Figure 2). Full Blood Count revealed haemoglobin of 5.8 g/L, white blood cell count of 10.1 × 10^3^/*μ*L, lymphocytes of 46.4%, neutrophils of 47.7%, and erythrocyte sedimentation rate of 105 mm/hour. Both pus from the pleural aspirate and wound swab culture grew* Proteus* spp. sensitive to quinolones and ceftriaxone. Pus Ziehl-Neelsen stains revealed no acid fast bacilli and Mantoux test was nonreactive. He initially had intravenous crystalline penicillin and intramuscular gentamycin which was later changed to quinolones based on the antimicrobial sensitivity for 6 weeks. He was also commenced on frusemide, antituberculous drugs, and nasogastric tube feeding and transfused with packed red blood cells. Patient was comanaged with surgeons who inserted chest tube for drainage and the child had clinical and radiological improvement after 2 weeks of treatment (Figure 3). Patient was discharged after 3 weeks of admission and followed up by the managing paediatric doctors. Patient was finally referred to the cardiothoracic surgeons for further management.

Management of this case was challenging in terms of diagnosis and treatment. Diagnosis of tuberculosis in this case was based on history only since investigation did not support the diagnosis. Low diagnostic yield of gastric aspirate for acid fast bacilli and negative Mantoux test due to anergy associated with malnourished children make it difficult to diagnose tuberculosis in this case. Contrast enhanced computed tomographic (CECT) scan which is the diagnostic study of choice that will show lung and mediastinal windows and reveal the extent and nature of the disease was not available. The isolation of* Proteus* species from the pleural fluid aspirate and wound swab suggests* Proteus* as the etiologic agent of the parapneumonic effusion. The dramatic resolution of symptoms in this case with anti-*Proteus* antibiotics could also suggest empyema necessitans complicating pleural effusion secondary to* Proteus* species.

## 3. Discussion

Pleural effusion with empyema necessitans is a cause of morbidity and mortality in children. It is characterised by pus collection in the thorax which bursts and communicates with the exterior, forming a fistula between the pleural cavity and the skin [1]. Empyema necessitans complicating pleural effusion is rare in our environment. This was the first case seen in our hospital for the past 12 years confirming the rarity of the condition. It is also reported to be rare by other workers elsewhere [2, 4]. Akgül et al. [2] reported only nine cases of empyema necessitans over a 4-year period in Turkey. Hoffman [5], in United Kingdom, also reported its rarity where he reported a prevalence of 3.2% (4/125). This patient's empyema and chest wall swelling were present for three months before presenting to our hospital for intervention. If pleural effusion is left for several months without intervention, this can lead to developing this complication, empyema necessitans [6, 7]. This might have contributed to the development of empyema necessitans in our patient.

The isolation of* Proteus* species from the pleural fluid in our patient indicates that this condition is probably due to the isolated organisms. This is in conformity with the reports by some workers [7, 8] who documented the etiologic agents to be Gram negative bacilli,* Streptococcus pneumoniae*,* Staphylococcus aureus,* and Blastomycosis. This finding contrasted with the reports by others where they documented more indolent pathogens,* Mycobacterium tuberculosis* and* Actinomyces israelii*, as a common cause of empyema necessitans [6]. Our finding also contrasted with the report [4] that most cases occur in immunocompromised patients because our case was seronegative for HIV. Our patient might be immunocompromised since he was severely malnourished.

Management of this case was challenging as this case was malnourished and features of TB may not be prominent. It was only the chest X-ray that suggested TB. Other investigations like Mantoux test, sputum, and pleural pus AFB were not diagnostic of tuberculosis. Differentiating tuberculous from nontuberculous empyema was very difficult because of low diagnostic yield of gastric aspirate for acid fast bacilli. Furthermore, malnutrition in children may suppress the tuberculin sensitivity leading to a negative Mantoux test which explains the difficulty in diagnosing tuberculosis in this case.

This patient's diagnosis was based on clinical, chest X-rays, and pleural fluid and wound swab microscopy culture and sensitivity. Contrast enhanced CT (CECT) scan was not done due unavailability of the facilities. Studies [8, 9] also revealed that the majority of empyema thoracis studied was based on a chest radiograph and not on a CT scan as was the case in our report. This may lead to incorrect judgment of the stage of the disease as well as delay in surgical intervention posing a challenge in managing the patient. However, a chest radiograph will only show opacity occupying a certain area of the hemithorax, which may be secondary to consolidated parenchyma, pleural peel, or a lung abscess. The CECT scan is the diagnostic study of choice with lung and mediastinal windows and reveals the extent and nature of the disease like demonstrating a communication of empyema into subcutaneous tissue [3, 8–10]. However, chest CECT could not be done in many centres, including ours, due to lack of facilities in most developing countries.

Early diagnosis and management of pleural effusion would prevent the development of empyema necessitans but our patient was not diagnosed and managed early necessitating the development of this complication [11]. The management consists of antimicrobials, tube drainage, and decortication for obliterating the cavity to prevent fibrosis and facilitate lung expansion [11]. Our case had antimicrobials therapy, tube drainage, and nutritional rehabilitation and was referred to the cardiothoracic surgeons for other management.

## 4. Conclusion

Empyema necessitans is a rare complication of pleural space infection. It is commonly associated with pulmonary tuberculosis,* Actinomyces*, and nontuberculous organisms like* Staphylococcus aureus*. Pulmonary infections with Gram negative organisms like* Proteus *spp. should also be considered as a cause of pleural effusion with empyema necessitans. The management of this case was challenging since it was difficult to differentiate between tuberculous and nontuberculous effusion in this case.
